# Basic Helix-Loop-Helix Transcription Factors AabHLH2 and AabHLH3 Function Antagonistically With AaMYC2 and Are Negative Regulators in Artemisinin Biosynthesis

**DOI:** 10.3389/fpls.2022.885622

**Published:** 2022-06-06

**Authors:** Qian Shen, Huayi Huang, Lihui Xie, Xiaolong Hao, Sadaf-Ilyas Kayani, Hang Liu, Wei Qin, Tiantian Chen, Qifang Pan, Pin Liu, Kexuan Tang

**Affiliations:** ^1^Plant Biotechnology Research Center, SJTU–Fudan–Nottingham Plant Biotechnology R&D Center, School of Agriculture and Biology, Shanghai Jiao Tong University, Shanghai, China; ^2^Laboratory of Medicinal Plant Biotechnology, College of Pharmacy, Zhejiang Chinese Medical University, Hangzhou, China

**Keywords:** bHLH, MYC2, transcription regulation, *Artemisia annua*, artemisinin

## Abstract

Plants have evolved sophisticated systems for regulating the biosynthesis of specialized phytochemicals. Artemisinin, which is a sesquiterpene lactone widely used in anti-malaria treatment, is produced by the *Artemisia annua* L. plant. However, the artemisinin content in *A. annua* is low and difficult to meet market demands. Studies have shown that artemisinin biosynthesis in *A. annua* has complex temporal and spatial specificity and is under tightly transcriptional regulation. However, the mechanism of transcriptional regulation of artemisinin biosynthesis remains unclear. In this study, we identified two MYC-type bHLH transcription factors (AabHLH2 and AabHLH3) as novel regulators of artemisinin biosynthesis. These bHLH TFs act as transcription repressors and function redundantly to negatively regulate artemisinin biosynthesis. Furthermore, AabHLH2 and AabHLH3 are nuclear proteins that bind to DNA elements with similar specificity to that of AaMYC2, but lack the conserved activation domain, suggesting that repression is achieved by competition for the same *cis*-regulatory elements. Together, our findings reveal a novel artemisinin biosynthesis regulatory network, provide new insight into how specialized metabolites are modulated in plants, and propose a model in which different bHLH TFs coordinated in regulating artemisinin production in the plant. Finally, this study provides some useful target genes for metabolic engineering of artemisinin production via CRISPR/Cas9 gene editing.

## Introduction

Artemisinin, a sesquiterpene lactone, extracted from the Chinese traditional medicinal plant *Artemisia annua* L, is the main component of artemisinin-based combinatory therapies (ACT) to treat malaria. Besides the antimalarial activities, artemisinin is also a multi-functional compound that has demonstrated cytotoxicity against cancer (Tin et al., 2012), schistosomiasis (Utzinger et al., 2007), virus (Obeid et al., 2013), and tuberculosis (Zheng et al., 2017). Due to the important medicinal properties of artemisinin, understanding how the artemisinin biosynthetic pathway is regulated is of extreme importance. Despite the great achievement in producing artemisinin in microbes via semisynthetic synthesis (Ro et al., 2006; Paddon et al., 2013), the *A. annua* plant is still the most important resource for artemisinin (Peplow, 2016). However, the transcriptional regulation of this important pathway in *A. annua* is not yet well established.

The family of bHLH TFs is very widespread among eukaryotes and exists in plants, animals, and fungi (Feller et al., 2011). The bHLH family consists of an N-terminal stretch of basic amino acid residues responsible for DNA (Goossens et al., 2017), which recognizes the E-box sequences (CANNTG) in the promoter of their target genes (Ezer et al., 2017). It has been found that bHLH TFs play critical regulatory roles in the specialized metabolism of medicinal and crop species. In Arabidopsis, AtMYC2, AtMYC3, and AtMYC4 were reported to regulate some flavonoid compounds biosynthesis (Dombrecht et al., 2007; Schweizer et al., 2013). The bHLH members of CrMYC2, CrBIS1, and CrBIS2 were involved in monoterpene indole alkaloids biosynthesis in the *Catharanthus roseus* plant (Zhang et al., 2011; Van Moerkercke et al., 2015, 2016). The NbbHLH1 and NbbHLH2 were found to regulate the pyridine alkaloids biosynthesis in *Nicotiana benthamiana* (Todd et al., 2010). In the medical plant *Salvia miltiorrhiza*, the SmMYC2a and SmMYC2b participated in the transcriptional regulation of tanshinones and phenolic acids biosynthesis (Zhou et al., 2016; Yang et al., 2017).

With nearly 200 predicted members in *A. annua*, the bHLH TFs comprise one of the largest TF families in the *A. annua* plant (Shen et al., 2018). To date, several bHLH TFs were reported that positively regulate the accumulation of artemisinin in *A. annua* under different conditions (Ji et al., 2014; Shen et al., 2016; Li et al., 2019; Xiang et al., 2019). Of these, the best characterized and most multifunctional are the MYC-type (MYC2 and AabHLH1) transcription factors, which are the members of the bHLH subgroup IIIe according to the classification by Heim et al. (2003). The MYC2 type of bHLH TFs are usually acting as the positive regulators in secondary metabolism in many plant species (Dombrecht et al., 2007; Todd et al., 2010; Zhang et al., 2011; Ji et al., 2014; Shen et al., 2016; Li et al., 2019; Xiang et al., 2019). On the contrary, JA-ASSOCIATED MYC2-LIKE1 (AtJAM1), AtJAM2, and AtJAM3 (AtbHLH17, −13, and −3, respectively) belong to the bHLH IIId subgroup in *A. thaliana*, function as transcription repressors to antagonize the transcription activator MYC2, and display negative regulation functions (Sasaki-Sekimoto et al., 2013; Song et al., 2013; Fonseca et al., 2014; Qi et al., 2015). Considering the important roles and complex network regulation mechanisms of MYC-type TFs in regulating plant secondary metabolism, it is interesting to ask whether there are other MYC-type bHLH TFs that regulate the biosynthesis of artemisinin.

To identify novel regulators of the artemisinin biosynthesis in the *A. annua* plant, gene co-expression analysis and phylogenetic analysis were performed. In the present study, we identified AabHLH2 and AabHLH3 as two new artemisinin biosynthesis regulators that show high similarity to AaMYC2, and their expression pattern is correlated with the artemisinin synthetic pathway genes. We then conducted overexpression and RNAi experiments to explore the functions of AabHLH2 and AabHLH3 in the *A. annua* plant. The overexpressed transgenic plants showed significantly lower accumulations of artemisinin, while the RNAi transgenic plants displayed higher artemisinin content when compared with the wild-type plants. Thus, we propose that AabHLH2 and AabHLH3 are novel factors that function mostly antagonistically to AaMYC2 in regulating the artemisinin metabolic pathway in *A. annua*. Our research indicates that the coordinated regulation of artemisinin by the transcription activators and repressors provides clues about the previously unknown complex mechanism for directing the production of secondary metabolites.

## Results

### Combining Phylogenetic Analysis and Global Gene Expression of bHLH Transcription Factors in *Artemisia annua*

The bHLH transcription factor family is one of the largest transcription factor families in the plant. However, up to now, there were only a few bHLH transcription factors, AabHLH1, AaMYC2, and AabHLH112, reported positively in regulating artemisinin biosynthesis in *A. annua* plant (Ji et al., 2014; Shen et al., 2016; Li et al., 2019; Xiang et al., 2019). Sequence analysis of those two genes showed that they both belong to the MYC-type bHLH transcription factor. MYC-type bHLH transcription factors extensively participate in the plant’s secondary metabolism process (Ramsay et al., 2003; Dombrecht et al., 2007; Hong et al., 2012; Schmiesing et al., 2016; Zhou et al., 2016). Therefore, further investigations on the regulatory functions of this sub-family member in *A. annua* are noteworthy. Benefited by the completion of the *A. annua* genome sequence, genome-wide analysis of MYC-type bHLH TFs in *A. annua* is feasible. The genes which encode putative MYC-type proteins were identified using the conserved MYC-type domain (Pfam: PF14215) through the HMM (Hidden Markov Model) search program. The output contains 45 genes that harbor the MYC-type domain. After manually checking the turnout results, sequences with low confidence (E value > 1 × 10^–10^) and redundancy are removed; therefore, a total of 35 unique genes encoding proteins containing the MYC-type domain were gained. In this study, we focused on the MYC-type bHLH transcription factors, therefore, by comparing with the bHLH transcription factor family in *A. annua*, it showed that 20 of the 35 candidate MYC-type genes belonged to the bHLH family.

**FIGURE 1:**
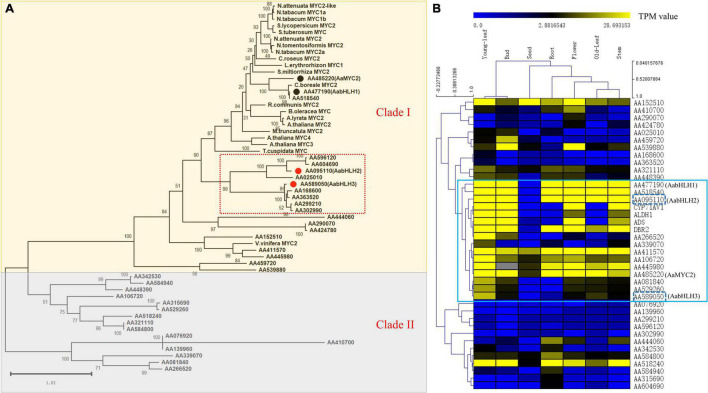
Phylogenetic analysis and global gene expression of MYC-type transcription factors in *A. annua*. **(A)** Phylogenetic tree analysis of MYCs transcription factors in *A. annua* with other plants MYCs. **(B)** Gene co-expression analysis of the MYC transcription factor subfamily with artemisinin biosynthetic pathway genes in *A. annua.*

To identify novel bHLH transcription factor regulators of the artemisinin biosynthesis, we performed a combined analysis of genome-wide phylogenetic analysis and gene expression profiles of different tissues via RNA-seq data. The transcriptomic sequencing data of seven different tissues of *A. annua* were generated by our lab before (Shen et al., 2018). To investigate whether, in addition to AabHLH1 and AaMYC2, other MYC-type bHLHs might contribute to the artemisinin biosynthesis of *A. annua*, phylogenetic analysis with characterized MYC-type in other plants was performed based on the clustering of their translated protein sequences in a Maximum Likelihood method. All the MYC-type genes were grouped into two distinct groups, the MYC-type bHLH group (Clade I) and the MYC-type non-bHLH group (Clade II) (Figure 1A). In Clade I, in which both AabHLH1 and AaMYC2 were grouped, there was a branch (highlighted in a red box) that was close to most of the MYC2-type transcription factors from different plants and with a specialty. There were nine MYC-type bHLH genes gathered on this branch, and to further understand the function of those genes, we launched the gene expression profiles analysis.

The result was shown as a hierarchical cluster analysis in a heatmap (Figure 1B) and showed that there were 12 MYC-type genes grouped with the specific artemisinin biosynthetic pathway genes (*ADS*, *CYP71AV1*, *DBR2*, and *ALDH1*) and demonstrated potential correlative with artemisinin biosynthesis. Together, combining the phylogenetic analysis and gene expression profile results indicated that there were two MYC-type bHLH genes, *AabHLH2* (AA095110) and *AabHLH3* (AA589050), which were located in the specific phylogenetic tree branch mentioned earlier, meanwhile showed co-expression pattern with pathway genes and demonstrated that they may have the potential roles in the regulation of artemisinin biosynthesis.

### Characterization of AabHLH2 and AabHLH3, and They Act as Transcriptional Repressors

The full-length coding regions of *AabHLH2* and *AabHLH3* correspond to open reading frames of 1,461 bp and 1,254 bp, respectively, and were isolated from cDNA prepared from leaves of the *A. annua* Huhao 1# cultivar. AabHLH2 shows a 63% sequence similarity to the *Helianthus annuus* (sunflower) MYC2-like transcription factor (Badouin et al., 2017) and AabHLH3 shows a 65% sequence similarity to the *Cynara cardunculus* MYC4-like transcription factor (Scaglione et al., 2016).

To determine where the AabHLH2 and AabHLH3 proteins functions within the cell, we determined the subcellular localization of AabHLH2 and AabHLH3 via fused with YFP protein. In contrast to YFP, which was distributed throughout all the cells, both AabHLH2-YFP and AabHLH3-YFP fusion proteins were observed exclusively in the nuclei (Supplementary Figure 1) of *N. benthamiana* leaf epidermal cells, suggesting that AabHLH2 and AabHLH3 are nuclear-localized proteins and this consistent with their potential function as transcriptional regulators.

**FIGURE 2:**
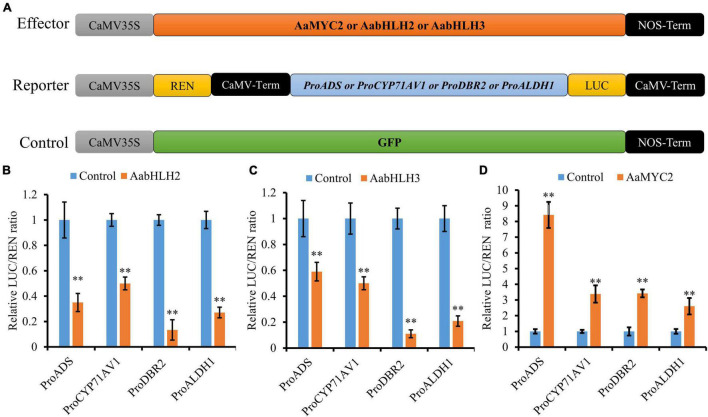
AabHLH2 and AabHLH3 regulate artemisinin biosynthetic genes expression as transcriptional repressors. **(A)** Schematic representation of effector and reporter constructs used in the Dual-LUC assays; **(B,C)** Transient Dual-LUC analysis of AabHLH2 and AabHLH3 with four artemisinin biosynthesis specific pathway gene promoters. **(D)** Transient Dual-LUC analysis of AaMYC2 (positive control) with four artemisinin biosynthesis specific pathway gene promoters; The values were reached by calculating the relative ratio of LUC activities to REN activities (LUC/REN) and the GFP control was set to one. Data are means ± SD (*n* = 4). Student’s *t*-test: *, *P* < 0.05; **, *P* < 0.01.

To further assess if AabHLH2 and AabHLH3 are regulators of artemisinin biosynthesis, we performed promoters (*ADS*, *CYP71AV1*, *DBR2*, and *ALDH1*) transactivation assays with AabHLH2 and AabHLH3 in *N. benthamiana* by Dual-LUC analysis (Figure 2A). To our surprise, when AabHLH2 and AabHLH3 were co-infiltrated in *N. benthamiana* leaf cells with proADS:LUC, proCYP71AV1:LUC, proDBR2:LUC, and proALDH1:LUC reporters, respectively, all the promoter activities were significantly decreased while comparing with the control set (Figures 2B,C). However, the AaMYC2 effector could upregulate the four aforementioned promoters’ activities, which is consistent with the previous report (Shen et al., 2016; Figure 2D). Together, these results indicated that AabHLH2 and AabHLH3 have transcriptional repression activities and may play as the negative regulators of artemisinin biosynthesis.

### Overexpression of *AabHLH2* or *AabHLH3* Decreases Artemisinin Content and Attenuated Expression of *AabHLH2* or *AabHLH3* Promotes Artemisinin Production in *Artemisia annua*

To further explore the physiological functions of AabHLH2 and AabHLH3 in *A. annua*, we generated transgenic *A. annua* plants, which include *AabHLH2* and *AabHLH3* overexpressing transgenic lines and *AabHLH2* and *AabHLH3* RNAi transgenic lines.

**FIGURE 3:**
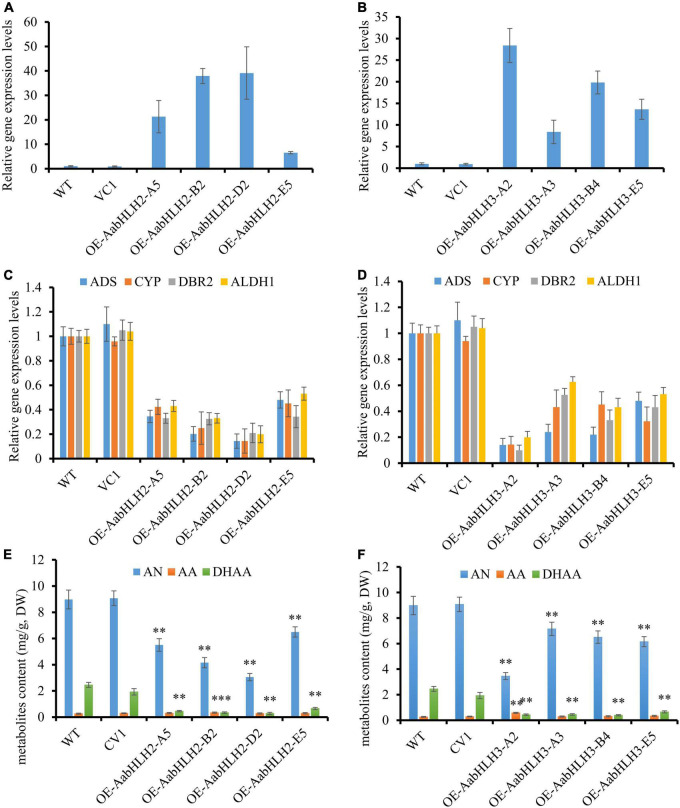
Overexpressing of *AabHLH2* and *AabHLH3* affects artemisinin biosynthetic pathway gene expression levels and artemisinin-related metabolites contents. **(A)** Gene expression analysis of *AabHLH2* in the *AabHLH2* overexpressing transgenic plants. **(B)** Gene expression analysis of *AabHLH3* in the *AabHLH3* overexpressing transgenic plants. **(C)** Relative gene expression of *ADS*, *CYP71AV1*, *DBR2*, and *ALDH1* in the *AabHLH2* overexpressing transgenic plants. **(D)** Relative gene expression of *ADS*, *CYP71AV1*, *DBR2*, and *ALDH1* in the *AabHLH3* overexpressing transgenic plants. **(E)** The contents of artemisinin (AN), dihydroartemisinic acid (DHAA), and artemisinic acid (AA) in the *AabHLH2* overexpressed plants and wild-type plants, determined by HPLC analysis. **(F)** The contents of artemisinin (AN), dihydroartemisinic acid (DHAA), and artemisinic acid (AA) in the *AabHLH3* overexpressed plants and wild-type plants, determined by HPLC analysis. The metabolites contents in the transgenic plant leaves were compared to the wild-type plants. Asterisks indicate the difference between overexpressing transgenic plants and wild-type plants. Statistical significance was determined by Student’s *t*-test: **, *P* < 0.01; *, *P* < 0.05.

The transgenic plants were confirmed by genomic PCR, and subsequently, qRT-PCR was used to analyze the transcript levels of *AabHLH2* and *AabHLH3* in the transgenic plants. The transcript levels of *AabHLH2* and *AabHLH3* were significantly higher in the overexpressed transgenic lines than in the WT and vector control transgenic plants (Figures 3A,B). As expected, the results were consistent with the previous Dual-LUC results that AabHLH2 and AabHLH3 inhibit the promoter activities of those pathway genes, and the expression levels of *ADS*, *CYP71AV1*, *DBR2*, and *ALDH1* were all dramatically decreased in *AabHLH2* and *AabHLH3* overexpression plants, respectively, compared with the WT or vector control plants (Figure 3D). This suggests that AabHLH2 and AabHLH3 have the potential role of negatively regulating artemisinin biosynthesis in *A. annua*. We performed high-performance liquid chromatography (HPLC) to analyze the relevant metabolites in the transgenic and WT plants. In line with the downregulation of genes involved in artemisinin biosynthesis, the HPLC results showed that the artemisinin (AN) and dihydroartemisinic acid (DHAA) contents in *AabHLH2* overexpression lines significantly reduced by 27 to 66% and 73 to 88%, respectively, compared with WT (Figure 3E), whereas the artemisinic acid (AA) content was not altered too much (Figure 3E) and the AN and DHAA contents in *AabHLH3* overexpression lines significantly reduced by 20 to 61% and 72 to 84%, respectively, compared with WT (Figure 3F), and also the AA content was not influenced too much (Figure 3F) (*P* < 0.05, Student’s *t*-test). No obvious phenotype differences were observed in the transgenic *A. annua* plants compared with the control vector (VC1) and WT plants. All these results indicate that AabHLH2 and AabHLH3 regulate artemisinin biosynthesis in the negative model.

**FIGURE 4:**
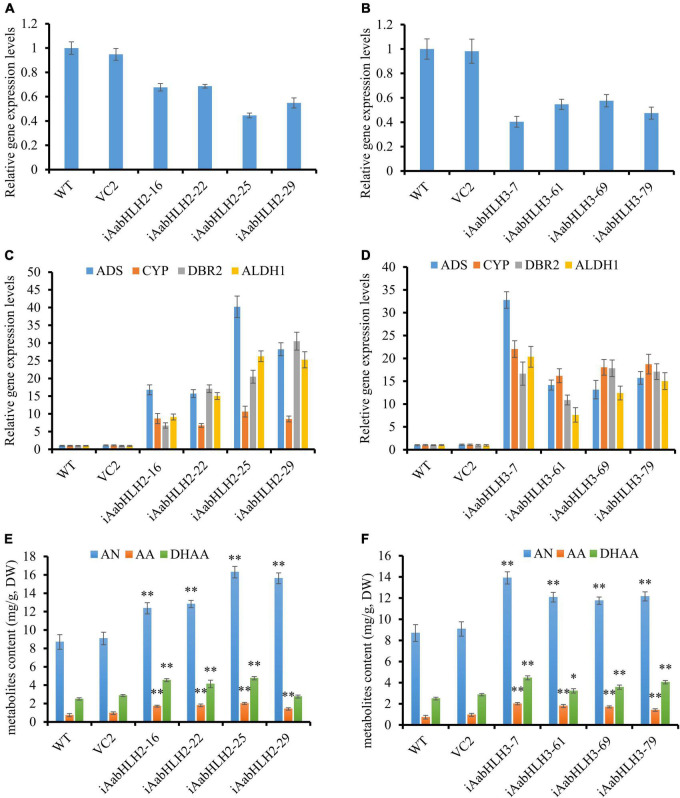
RNA interference reducing *AabHLH2* and *AabHLH3* gene expression affects artemisinin biosynthetic pathway gene expression levels and artemisinin-related metabolites contents. **(A)** Gene expression analysis of *AabHLH2* in the *AabHLH2* RNAi transgenic plants. **(B)** Gene expression analysis of *AabHLH3* in the *AabHLH3* RNAi transgenic plants. **(C)** Relative gene expression of *ADS*, *CYP71AV1*, *DBR2*, and *ALDH1* in the *AabHLH2* RNAi transgenic plants. **(D)** Relative gene expression of *ADS*, *CYP71AV1*, *DBR2*, and *ALDH1* in the *AabHLH3* RNAi transgenic plants. **(E)** The contents of artemisinin (AN), dihydroartemisinic acid (DHAA), and artemisinic acid (AA) in the *AabHLH2* RNAi plants and wild-type plants were determined by HPLC analysis. **(F)** The contents of artemisinin (AN), dihydroartemisinic acid (DHAA), and artemisinic acid (AA) in the *AabHLH3* RNAi plants and wild-type plants were determined by HPLC analysis. The metabolites contents in the transgenic plant leaves were compared to the wild-type plants. Asterisks indicate the difference between RNAi transgenic plants and wild-type plants. Statistical significance was determined by Student’s *t*-test: **, *P* < 0.01; *, *P* < 0.05.

To further verify the biological roles of AabHLH2 and AabHLH3 in controlling artemisinin biosynthesis, we silenced these genes’ expression by RNA interference. The transcript levels of *AabHLH2* and *AabHLH3* were significantly reduced in the RNAi transgenic lines while compared with the WT and vector control plants (Figures 4A,B). As expected, the expression levels of *ADS*, *CYP71AV1*, *DBR2*, and *ALDH1* were all upregulated in *AabHLH2* and *AabHLH3* overexpression plants, respectively, compared with the WT or vector control plants (Figures 4C,D). Consistent with this, the contents of AN, DHAA, and AA in *AabHLH2* RNAi plants were increased by 42 to 87%, 10 to 90%, and 93 to 175%, respectively, compared with the WT plants (Figure 4E). Similarly, the contents of AN, DHAA, and AA in *AabHLH3* RNAi plants were increased by 35 to 60%, 29 to 79%, and 65 to 156%, respectively, compared with the WT plants (Figure 4F; *P* < 0.05, Student’s *t*-test). No obvious differences were observed in *A. annua* plants that transformed with the control vector (VC2). Together, all the results indicate that AabHLH2 and AabHLH3 are the negative regulators of artemisinin biosynthesis and may be good targets in efforts to increase artemisinin production through CRISPR/Cas9 gene-editing knockout in *A. annua* plant. We also speculate that overexpressing *AaMYC2* and silencing/knockout *AabHLH2/3* simultaneously is a promising genetic engineering strategy to dramatically enhance concentrations of artemisinin.

### AabHLH2 and AabHLH3 Inhibit Artemisinin Biosynthesis *via* Antagonizing AaMYC2 for Target Genes

**FIGURE 5:**
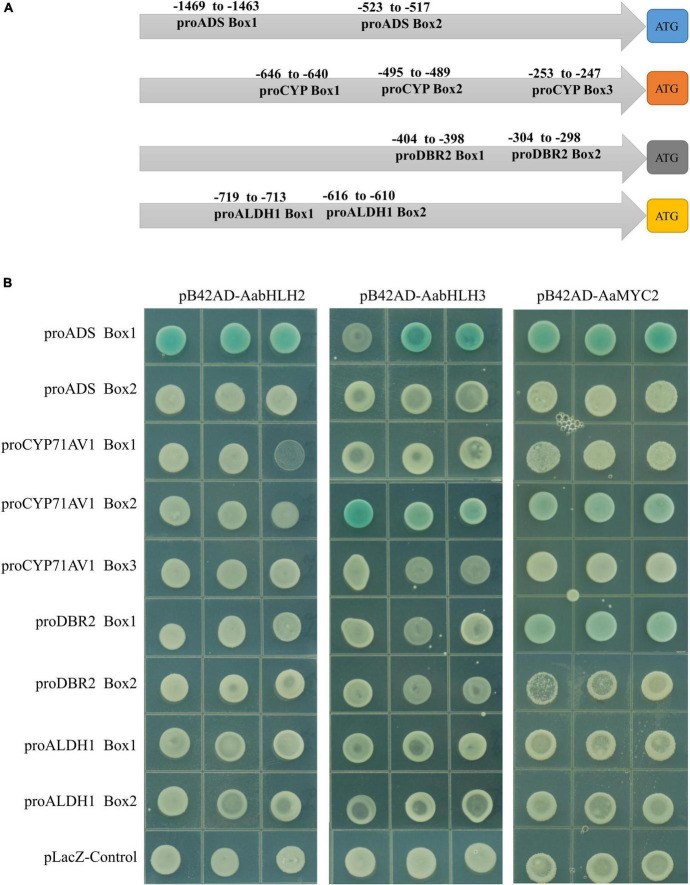
Binding assay of AabHLH2 and AabHLH3 to G-box-like *cis*-elements in the promoters via yeast one-hybrid. **(A)** Schematic representation of all G-box-like *cis*-elements boxes position in the four pathway gene promoters. **(B)** The result of yeast one-hybrid assay for the interaction between AabHLH2 and AabHLH3 with DNA motifs.

Sequence alignment revealed that the basic HLH domain is highly conserved between AabHLH2, AabHLH3, and AaMYC2, as well as other MYC-type bHLH proteins (Supplementary Figure 2). The basic HLH domain is mainly involved in DNA binding activities (Carretero-Paulet et al., 2010; Fernandez-Calvo et al., 2011; Goossens et al., 2017), and we hypothesized that AabHLH2 and AabHLH3 may bind to the same promoter regions as AaMYC2. Previous studies suggested that usually the bHLH family proteins directly bind to the E-box (CANNTG) element in the promoter sequence of target genes and MYC-type bHLH subfamily members prefer the G-box (CACGTG) element which is one type of the E-box elements (Atchley and Fitch, 1997; Atchley et al., 2000; Boter et al., 2004; Dombrecht et al., 2007). Promoter analysis revealed that there were two G-box-like motifs in the promoter of *ADS*, three G-box-like motifs in the promoter of *CYP71AV1*, two G-box-like motifs in the promoter of *DBR2*, and two G-box-like motifs in the promoter of *ALDH1* (Figure 5A). Yeast one-hybrid assay revealed that the pB42AD-AabHLH2 fusion protein binding to the three tandem repeats of the ADS-Box1 motif, while the pB42AD-AabHLH3 fusion protein binding to the three tandem repeats of the ADS-Box1 and CYP-Box2 motif, respectively (Figure 5B). Meanwhile, we also performed the Y1H assay of AaMYC2 and showed that AaMYC2 binding to the ADS-Box 1, CYP-Box2, and DBR2-Box1, respectively (Figure 5B). Together, the above results demonstrate that AabHLH2 and AabHLH3 have similar but not the same DNA binding preferences as AaMYC2.

**FIGURE 6:**
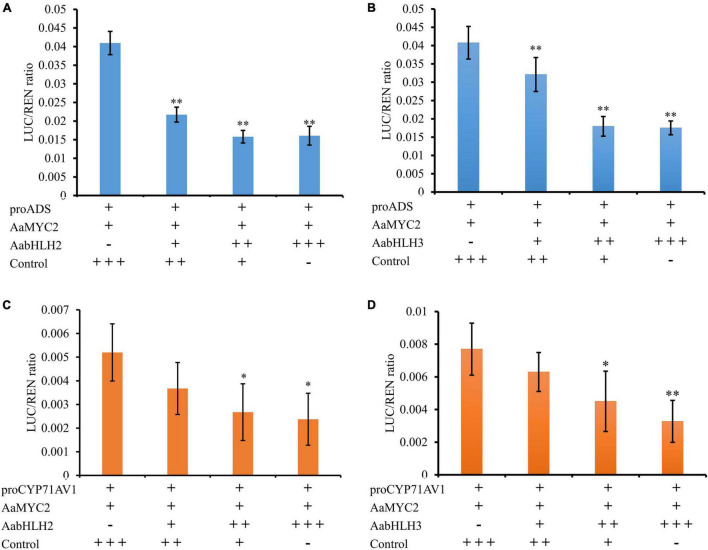
AabHLH2 and AabHLH3 were competing with AaMYC2 to the target gene promoters. **(A,B)** The competition effect of AabHLH2/AabHLH3 with AaMYC2 in regulating *ADS* promoter, the activation of the *ADS* promoter was attenuated in a dose-dependent manner when co-expressing of *AabHLH2*/AabHLH3 and *AaMYC2*, respectively. **(C,D)** The competition effect of AabHLH2/AabHLH3 with AaMYC2 in regulating the *CYP71AV1* promoter, the activation of the *CYP71AV1* promoter was attenuated in a dose-dependent manner when co-expressing of *AabHLH2*/AabHLH3 and *AaMYC2*, respectively. The values were reached by calculating the relative ratio of LUC activities to REN activities (LUC/REN), and the GFP was added as a control. Data are means ± SD (*n* = 4). Student’s *t*-test: *, *P* < 0.05; **, *P* < 0.01.

One of the possible mechanisms of AabHLH2 and AabHLH3 repressions of gene expression is that AabHLH2 and AabHLH3 may repress gene transcription competitively with MYC2 for target sequences. To test whether AabHLH2 and AabHLH3 proteins antagonize the binding of AaMYC2 to its target promoters, we performed the competition assays by transient expression of *AabHLH2*, *AabHLH3*, and *AaMYC2* with the *ADS* and *CYP71AV1* promoter reporters, respectively. The Dual-LUC analysis showed that AaMYC2 was able to induce the activity of *ADS* and *CYP71AV1* promoters dramatically (Figure 2D). However, the transcriptional activation of the proADS-regulated LUC by AaMYC2 was attenuated in a dose-dependent manner by co-expression of AabHLH2 or AabHLH3 (Figures 6A,B). The proCYP71AV1-regulated LUC activation showed a similar pattern (Figures 6C,D). The transient expression analysis using the LUC reporter revealed the competition effect of AabHLH2 or AabHLH3 with AaMYC2 in regulating target genes. Taken together, these data demonstrate that AabHLH2 and AabHLH3 proteins antagonize AaMYC2 by binding to its target gene (*ADS* and *CYP71AV1*) promoters and act as transcriptional repressors.

## Discussion

Malaria is still a global health problem, according to the WHO world malaria report 2021 (World Health Organization [WHO], 2021). Artemisinin, a sesquiterpene lactone, extracted from the Chinese traditional medicinal plant *A. annua*, is the main component of artemisinin-based combinatory therapies (ACT) to treat malaria. Although tremendous amount of energy has been invested in increasing the artemisinin content such as semi-synthetic via engineering yeast (Ro et al., 2006; Paddon et al., 2013), heterogenous production in other plants (van Herpen et al., 2010; Fuentes et al., 2016; Ikram and Simonsen, 2017), the *A. annua*-derived artemisinin is still the main source. Therefore, current artemisinin production by plants is still not sufficient to meet global needs for malaria treatment. Several TF regulators have reported that they are involved in artemisinin biosynthesis in the plant, but a more in-depth understanding of the underlying mechanism of transcriptional regulation is still unclear.

Commonly, plant secondary metabolites are precisely synthesized within the specific tissues at a certain time to coordinate the plant development and response to environmental issues, which are usually tightly controlled at the transcriptional level. This means that in one particular metabolic pathway, the enzyme-encoding genes, the transcription factors, the transporter encoding genes, and other regulator genes usually exhibit a concerted expression pattern (Goossens, 2014). A striking feature of the *A. annua* genome is the existence of a large gene number that may be caused by the expansion of the gene family. Therefore, for effective gene discovery in plant secondary metabolism, co-expression analysis has been proved to be a powerful tool (Goossens, 2014). For instance, the terpenoid indole alkaloids biosynthesis regulators CrORCA3, CrBIS1, CrBIS2, and CrERF5 in *C. roseus* (Zhang et al., 2011; Van Moerkercke et al., 2015, 2016) and the artemisinin biosynthesis regulators AabZIP9, AaGSW1 in *A. annua* were all discovered via gene co-expression analysis (Chen et al., 2017; Shen et al., 2019). On the other hand, in many cases, the transcription factors involving plant specialized metabolites regulation are conserved among different species. These TFs control the expression level of multiple biosynthetic enzyme genes of one specific pathway in a coordinated manner, resulting in the regulation of flux through the pathway (Van Moerkercke et al., 2015, 2016; Li et al., 2016; Goossens et al., 2017). Given their conserved role, these TFs can be considered potential candidates for metabolic engineering of plants and may be used in different plant species (Mertens et al., 2016; Shi et al., 2020). Consequently, fundamental research in model plants may transfers to other non-model plant species that produce specialized metabolites of interest for plants or humans.

The bHLH transcription factor family is one of the largest families in the plant. By combining the gene co-expression and phylogenetic analysis strategies, we demonstrated that two MYC-type bHLH TFs candidates (AabHLH2 and AabHLH3) displayed similar gene expression patterns with the artemisinin biosynthetic pathway genes closely clustered with all the other plant MYC2-type transcription factors (Figure 1B). Transactivation assays of AabHLH2 and AabHLH3 in *N. benthamiana* by Dual-LUC analysis revealed that AabHLH2 and AabHLH3 were both negatively regulating the transcriptional activity of the pathway genes promoters (Figures 2B,C). Our results demonstrated that gene co-expression analysis is not only useful for positive regulators discovery but also suitable for finding negative regulators.

**FIGURE 7:**
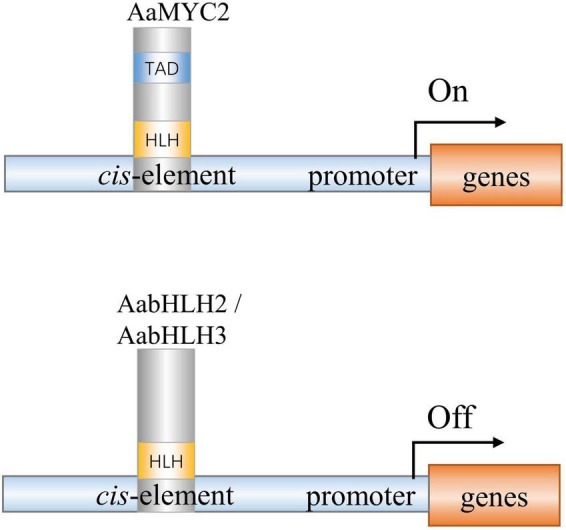
A simplified model of action of AabHLH2, AabHLH3, and AaMYC2 in regulating artemisinin biosynthesis in *Artemisia annua*. AabHLH2, AabHLH3, and AaMYC2 were all able to bind the G-box-like motifs in the pathway gene promoters. AaMYC2 contains the conserved TAD domain and activates the transcription of the downstream gene. AabHLH2 and AabHLH3 were lacking the conserved TAD domain and repressing the transcription of the downstream genes.

AabHLH2 and AabHLH3 have higher homology to jasmonate-associated MYC2-like proteins (AtJAMs) 1 to 3, rather than AtMYC2 or AaMYC2 (Supplementary Figure 2). AaMYC2 and AtMYC2 are categorized as IIIe group of bHLH TFs, while AabHLH2, AabHLH3, and AtJAMs are categorized as IIId group of bHLH TFs (Goossens et al., 2017). It was reported in Arabidopsis that the bHLH subgroup IIId TFs, including AtJAM1/2/3, function as transcription repressors to antagonize the transcription activator MYC2 (Sasaki-Sekimoto et al., 2013; Song et al., 2013; Qi et al., 2015). Unlike the MYC2-types TFs, the IIId group TFs lacks a transcriptional activation domain (TAD) and fail to recruit the RNA polymerase II complex such as MED25 to activate transcription (An et al., 2017; Liu et al., 2019; Wang et al., 2019). In this study, we also found that AabHLH2 and AabHLH3 have a similar binding ability to AaMYC2, which likely provides the mechanism for negative regulation of artemisinin biosynthesis (Figure 7). The antagonistic functions between the bHLH subgroup IIId members (e.g., JAM1,2,3) and IIIe members (e.g., MYC2,3,4) appear to be a more general mechanism of the balanced output of plant secondary metabolites biosynthesis.

Notably, there were several MYC-type non-bHLH genes clustered in the clade II branch that also showed similar gene expression patterns with the artemisinin biosynthetic pathway genes, such as AA081840, AA266520, and AA529260 (Figure 1). It will be interesting and worthwhile to investigate their functions whether they are positive regulators or negative regulators of artemisinin biosynthesis or maybe they are not involved in artemisinin biosynthesis regulation and have other functions.

Moreover, up to date, more and more artemisinin positive regulators were reported and only a few artemisinin negative regulators were investigated. With the development of gene-editing technology, the negative regulators are becoming more and more important. Our results indicate that AabHLH2 and AabHLH3 are the negative regulators of artemisinin biosynthesis and may be good targets in efforts to increase artemisinin production through CRISPR/Cas9 gene editing in *A. annua*. Screening for negative regulators suitable for gene editing to enhance artemisinin content may be a new strategy to improve the concentration of artemisinin in the plant.

## Materials and Methods

### Plant Material

The seeds of *A. annua* (cultivar “Huhao 1”) were bred by our lab for several years in Shanghai, China (Shen et al., 2016, 2018). Aseptic seedlings for genetic transformation were acquired according to the method reported previously. Seeds were surface-sterilized with 75% ethanol for 1 min and then treated using 20% (v/v) NaOCl for 10 min, followed by washing with sterilized water four times (Shen et al., 2016). The *Nicotiana benthamiana* (a close relative of tobacco) seeds were sown in pots and grown in a growth chamber under standard conditions of light, temperature, and humidity (16/8 light/dark period, 25 ± 2°C, 50%–70% relative humidity).

### Gene Discovery and Bioinformatics Analysis

To search for the MYC-type subfamily in *A. annua*, the conserved MYC-type domain (Pfam: PF14215) was downloaded from the Pfam database.^1^ The MYC-type homologs were identified through the HMM (Hidden Markov Model) search against the *A. annua* protein sequence database. Sequences with low confidence (*E* value >1× 10^–10^) and redundancy of the turnout results were then manually removed. The protein sequences of MYC2 type in other plants (retrieved from NCBI) and protein sequences of all the MYC-type in *A. annua* were aligned with ClustalW (Larkin et al., 2007). The non-rooted phylogenetic tree was generated with the MEGA6.1 software (Tamura et al., 2013), according to the Maximum Likelihood method with JTT matrix-based model and bootstrapping with 500 replicates to evaluate the accuracy of phylogenetic construction. The expression levels of each MYC-type TFs in seven different tissues (young leaf, old leaf, flower, bud, root, stem, and seed) were obtained by transcripts per million (TPM) normalization method as reported previously (Shen et al., 2018). Gene co-expression analysis was performed using the MultiExperiment Viewer (MeV4.9.0) software (Saeed et al., 2003) based on the TPM value of each gene. Sample clustering was carried out using the hierarchical clustering method, and the evolutionary distances were computed with Poisson correction (Eisen et al., 1998).

### Cloning of the Candidate MYC-Type bHLH Genes

For gene isolation, the gene-specific primers, AabHLH2 F/R and AabHLH3 F/R, were designated according to assembled RNA-seq data generated by our lab before (Shen et al., 2018). PCR was performed according to the manufacturer’s instructions for KOD DNA polymerase (Toyobo, Japan), using *A. annua* young leaf cDNA as the template. The PCR products were subcloned into the pLB vector (Tiangen, China) and confirmed by sequencing. All the primers used in the present study are listed in Supplementary Table 1.

### Dual-Luciferase Assay

To obtain plant overexpression vectors and perform the dual-luciferase assay (Dual-LUC), AabHLH2 and AabHLH3 ORF were amplified with primers P1 F/R and P2 F/R, and then cloned into pHB vector, respectively. The four reporter constructs were previously generated by our lab (Hao et al., 2017), which were produced by inserting the promoters of *ADS*, *CYP71AV1*, *DBR2*, and *ALDH1* into the pGreenII 0800-LUC plasmid (Hellens et al., 2005), respectively. The effector and reporter plasmids were transferred into the *A. tumefaciens* strain GV3101, respectively. The effector strain and reporter strain were mixed at the ratio 1:1, then the bacteria mixtures were transiently infiltrated into the *N. benthamiana* leaves as described previously (Shen et al., 2019). The effector strain that harbored pHB-GFP plasmid was used as the control. The firefly LUC and REN activities were analyzed using commercial Dual-LUC reaction reagents (Promega, United States) according to the manufacturer’s instructions. Four biological replicates were measured for each sample.

### Subcellular Localization Analysis

For subcellular localization analysis, AabHLH2 and AabHLH3 ORF were amplified with primers P3 F/R and P4 F/R and cloned into pENTR-SD/TOPO gateway vector (Invitrogen, United States) and subsequently recombined into pEarleyGate104 (Earley et al., 2006) by LR reaction (Invitrogen, United States) to generate YFP fusion vectors, pEarleyGate104-YFP-AabHLH2 and pEarleyGate104-YFP-AabHLH3, respectively. Subsequently, the above-mentioned plasmids were transferred into the *A. tumefaciens* strain GV3101 and transiently infiltrated into the *N. benthamiana* leaves as described previously (Shen et al., 2019). The YFP signals were observed after 3 days of cultivation using confocal microscopy (Leica, Germany), with argon laser excitation at 488 nm and a 505- to 550-nm emission filter set. Subcellular localization was done in three biological replicates.

### Yeast One-Hybrid

For yeast one-hybrid experiments, the full-length coding sequences of AabHLH2 and AabHLH3 were amplified with primers P7 F/R and P8 F/R and then cloned into the pB42AD (*Eco*RI/*Xho*I) by using ClonExpress II (Vazyme, China) to generate the effector vectors. The artificial synthesized triplicate *cis*-element segments contain possible bHLH binding elements from the promoters of *ADS*, *CYP71AV1*, *DBR2*, and *ALDH1*, which were inserted into the pLacZ (*Eco*RI/*Xho*I) to create the reporter vectors. The sequences of artificially synthesized triplicate *cis*-elements were listed in Supplementary Table 1. The combinations of pB42AD-AabHLH2 and pB42AD-AabHLH3 with different *cis*-element motifs were co-transformed into the yeast strain EGY48 by using the LiAc method. The transformant yeasts were cultivated on synthetic-defined SD/-Trp/-Ura dropout (Clontech, Dalian) selected plates at 30°C and positive clones were transferred to and grown on SD/-Trp/-Ura plates with X-gal to test the interaction.

### RNA Isolation and qPCR Analysis

Young leaves from 3-month-old transgenic and wild-type plants were harvested for gene expression analysis. Leaf samples were picked and immediately frozen in liquid nitrogen and stored at –80°C. The total RNA was extracted by RNAprep Pure Plant Kit (Tiangen, China) following the manufacturer’s instructions. 1 μg of total RNA was used for first-strand cDNA synthesis by using PrimeScript RT Master Mix Kit (TaKaRa, Dalian). qRT-PCR experiments were performed by using SuperReal PreMix SYBR Green Kit (Tiangen, China) on the LightCycle96 machine (Roche, Switzerland) as reported previously (Shen et al., 2016). The relative expression levels of genes were normalized to the expression of the *A. annua* β-ACTIN gene and calculated by the 2–ΔΔ*^Ct^*. All the primers used for qPCR are listed in Supplementary Table 1.

### Plant Transformation of *Artemisia nnua* and Genomic DNA PCR Analysis

To construct RNAi vector, approximately 400 bp length specific sequence of AabHLH2 and AabHLH3 is amplified with primers P5 F/R and P6 F/R and cloned into pENTR-SD/TOPO gateway vector (Invitrogen, United States) and is subsequently recombined into pHellsagate (Helliwell and Waterhouse, 2003) by LR reaction (Invitrogen, United States) to generate pHellsagate-iAabHLH2 and pHellsagate-iAabHLH3 vectors. The plant overexpression, RNAi, and control constructs (pHB-AabHLH2, pHB-AabHLH3, pHellsagate-iAabHLH2, pHellsagate-iAabHLH3, pHB-GUS, and pHellsagate) were introduced into *Agrobacterium tumefaciens* strain EHA105, respectively, and the resulting strains were used in the transformation of *A. annua*. The transgenic plants of *A. annua* were generated as described previously (Shen et al., 2012). To detect the positive transgenic plants, the genomic DNA was extracted from the *A. annua* plants by the CTAB method. The T_0_ transgenic plants of overexpression *A. annua* were confirmed by genomic DNA-based PCR on both inserted gene and Hyg (Hygromycin) resistant gene. For RNAi transgenic plant confirmation, both the inserted fragment and NPT II (kanamycin) resistance gene were analyzed by PCR. All the primers used are listed in Supplementary Table 1

### Quantification of Metabolites Using High-Performance Liquid Chromatography

Three-month-old plant leaves were harvested for HPLC analysis, and leaf samples of the transgenic plants and control plants were prepared as described previously (Lu et al., 2013). The leaves were dried in a drying oven at 45°C for 2 days and then ground into powder. A 0.1 g dried leaf powder of each sample was extracted with 2 ml methanol in an ultrasonic processor under the conditions of 25°C and 50W for 30 min. The samples were centrifuged for 10 min at 10,000 g and then the supernatants were passed through a 0.25-μm membrane filter. The filtrated solutions were then used for metabolites analysis via HPLC. The conditions for HPLC were set as described previously (Lu et al., 2013). Standard of artemisinin was purchased from Sigma and standard of dihydroartemisinic acid and artemisinic acid were bought from Guangzhou Honsea Sunshine BioScience and Technology Co. Ltd. (Honsea Sunshine Bio, China). Three biological replicates were measured for each sample.

## Data Availability Statement

The original contributions presented in the study are included in the article/Supplementary Material, further inquiries can be directed to the corresponding author.

## Author Contributions

QS and KT designed the project. QS and HH performed most of the experiments and wrote the manuscript. LX, XH, and S-IK performed some of the experiments. HL, WQ, TC, and QP analyzed the data and discussed the article. PL performed the HPLC analysis. All authors have read and approved the manuscript.

## Conflict of Interest

The authors declare that the research was conducted in the absence of any commercial or financial relationships that could be construed as a potential conflict of interest.

## Publisher’s Note

All claims expressed in this article are solely those of the authors and do not necessarily represent those of their affiliated organizations, or those of the publisher, the editors and the reviewers. Any product that may be evaluated in this article, or claim that may be made by its manufacturer, is not guaranteed or endorsed by the publisher.

## Footnotes

1
https://pfam.xfam.org/
