# Supplementary material for: Basic Helix-Loop-Helix Transcription Factors AabHLH2 and AabHLH3 Function Antagonistically With AaMYC2 and Are Negative Regulators in Artemisinin Biosynthesis
Source: Front Plant Sci. 2022 Jun 6;13:885622. doi: 10.3389/fpls.2022.885622 (PMC9207477; doi:10.3389/fpls.2022.885622)
Supplement: Supplementary file 1 [file Table_1.DOCX]

**Table S1 Primers used in this study**

| Primers | Purpose | Primer Sequence (5’-3’) |
| --- | --- | --- |
| AabHLH2F | Clone | AGGTTTTCTCACTCGTTTATTTC |
| AabHLH2R | Clone | TAGAGATAAAGATCGTTGAGATG |
| AabHLH3F | Clone | TATAAAACATGCGGCATTATTGC |
| AabHLH3R | Clone | TCACTATTACGTCATTCCACCAC |
| P1F | bHLH2 OE vector construct | CTCTCTCTAAGCTTGGATCCATGGATGATGATTTCCTAATTC |
| P1R | bHLH2 OE vector construct | GATACGAACGAAAGCTCTAGATTACTGATTTAATCTAGCTAGAAGATG |
| P2F | bHLH3 OE vector construct | CTCTCTCTAAGCTTGGATCCATGTTATTACTAAACTCAACC |
| P2R | bHLH3 OE vector construct | GATACGAACGAAAGCTCTAGATTAATCCAAGCACATTTTGTTG |
| P3F | bHLH2 pTOPO gateway vector | CACCATGGATGATGATTTCCTAATTC |
| P3R | bHLH2 pTOPO gateway vector | TTACTGATTTAATCTAGCTAGAAGATG |
| P4F | bHLH3 pTOPO gateway vector | CACCATGTTATTACTAAACTCAACC |
| P4R | bHLH3 pTOPO gateway vector | TTAATCCAAGCACATTTTGTTG |
| P5F | bHLH2 pTOPO gateway for RNAi | CACCACTCCAAGCCACGAATAACCAACC |
| P5R | bHLH2 pTOPO gateway for RNAi | GAGATGCTAAATTACATGACATGAT |
| P6F | bHLH3 pTOPO gateway for RNAi | CACCGCTTGAAGTCGAGGTGAAGTTAC |
| P6R | bHLH3 pTOPO gateway for RNAi | TCCCACCACTTTAGATGCTGATAC |
| P7F | bHLH2 pB42AD vector construct | GATTATGCCTCTCCCGAATTCATGGATGATGATTTCCTAATTC |
| P7R | bHLH2 pB42AD vector construct | GAAGTCCAAAGCTTCTCGAGTTACTGATTTAATCTAGCTAGAAGATG |
| P8F | bHLH3 pB42AD vector construct | GATTATGCCTCTCCCGAATTCATGTTATTACTAAACTCAACC |
| P8R | bHLH3 pB42AD vector construct | GAAGTCCAAAGCTTCTCGAGTTAATCCAAGCACATTTTGTTG |
| RT-AabHLH2 F | Real time PCR | CGCCTCCCGCTAATAATATGGC |
| RT-AabHLH2 R | Real time PCR | GGCAATCAGAATCCGACTCCTC |
| RT-AabHLH2 F | Real time PCR | TGTTTGATCCGAACAGCTTACC |
| RT-AabHLH2 R | Real time PCR | ATGTGTTCTTGGTTGTTACTG |
| RT-ADS F | Real time PCR | AATGGGCAAATGAGGGACAC |
| RT-ADS R | Real time PCR | TTTCAAGGCTCGATGAACTATG |
| RT-CYP F | Real time PCR | CGAGACTTTAACTGGTGAGATTGT |
| RT-CYP R | Real time PCR | CGAAGCGACTGAAATGACTTTACT |
| RT-DBR2 F | Real time PCR | GCGGTGGTTACACTAGAGAACTT |
| RT-DBR2 R | Real time PCR | ATAATCAAAACTAGAGGAGTGACCC |
| RT-ALDH1 F | Real time PCR | TGAGCCTACTCTATTTACAAACG |
| RT-ALDH1 R | Real time PCR | TAACAGTTGACCCAAACAGCA |
| RT-Actin F | Real time PCR | CCAGGCTGTTCAGTCTCTGTAT |
| RT-Actin R | Real time PCR | CGCTCGGTAAGGATCTTCATCA |
| proADS Box1F | Y1H placZ vector | aattcTTAGGTCACGTCTTAATTTAGGTCACGTCTTAATTTAGGTCACGTCTTAAT |
| proADS Box1R | Y1H placZ vector | tcgagATTAAGACGTGACCTAAATTAAGACGTGACCTAAATTAAGACGTGACCTAAg |
| proADS Box2F | Y1H placZ vector | aattcCTATCACACGTTAGAAGCTATCACACGTTAGAAGCTATCACACGTTAGAAGc |
| proADS Box2R | Y1H placZ vector | tcgagCTTCTAACGTGTGATAGCTTCTAACGTGTGATAGCTTCTAACGTGTGATAGg |
| proCYP Box1F | Y1H placZ vector | aattcCTAACACACGTATAGCACTAACACACGTATAGCACTAACACACGTATAGCAc |
| proCYP Box1R | Y1H placZ vector | tcgagTGCTATACGTGTGTTAGTGCTATACGTGTGTTAGTGCTATACGTGTGTTAGg |
| proCYP Box2F | Y1H placZ vector | aattcTCTTTTACGTGTCAATTTCTTTTACGTGTCAATTTCTTTTACGTGTCAATTc |
| proCYP Box2R | Y1H placZ vector | tcgagAATTGACACGTAAAAGAAATTGACACGTAAAAGAAATTGACACGTAAAAGAg |
| proCYP Box3F | Y1H placZ vector | aattcCCAAACCACGTAAGTTTCCAAACCACGTAAGTTTCCAAACCACGTAAGTTTc |
| proCYP Box3R | Y1H placZ vector | tcgagAAACTTACGTGGTTTGGAAACTTACGTGGTTTGGAAACTTACGTGGTTTGGg |
| proDBR2 Box1F | Y1H placZ vector | aattcAATTAAACGTGAAAAGTAATTAAACGTGAAAAGTAATTAAACGTGAAAAGTc |
| proDBR2 Box1R | Y1H placZ vector | tcgagACTTTTCACGTTTAATTACTTTTCACGTTTAATTACTTTTCACGTTTAATTg |
| proDBR2 Box2F | Y1H placZ vector | aattcTGTGATACGTGATATTATGTGATACGTGATATTATGTGATACGTGATATTAc |
| proDBR2 Box2R | Y1H placZ vector | tcgagTAATATCACGTATCACATAATATCACGTATCACATAATATCACGTATCACAg |
| proALDH1 Box1F | Y1H placZ vector | aattcGCACGCCACGTATGTATGCACGCCACGTATGTATGCACGCCACGTATGTATc |
| proALDH1 Box1R | Y1H placZ vector | tcgagATACATACGTGGCGTGCATACATACGTGGCGTGCATACATACGTGGCGTGCg |
| proALDH1 Box2F | Y1H placZ vector | aattcTTCTATCACGTAACCAATTCTATCACGTAACCAATTCTATCACGTAACCAAc |
| proALDH1 Box2R | Y1H placZ vector | tcgagTTGGTTACGTGATAGAATTGGTTACGTGATAGAATTGGTTACGTGATAGAAg |
|  |  |  |
