# Supplementary figures and images for: Basic Helix-Loop-Helix Transcription Factors AabHLH2 and AabHLH3 Function Antagonistically With AaMYC2 and Are Negative Regulators in Artemisinin Biosynthesis
Source: Front Plant Sci. 2022 Jun 6;13:885622. doi: 10.3389/fpls.2022.885622 (PMC9207477; doi:10.3389/fpls.2022.885622)

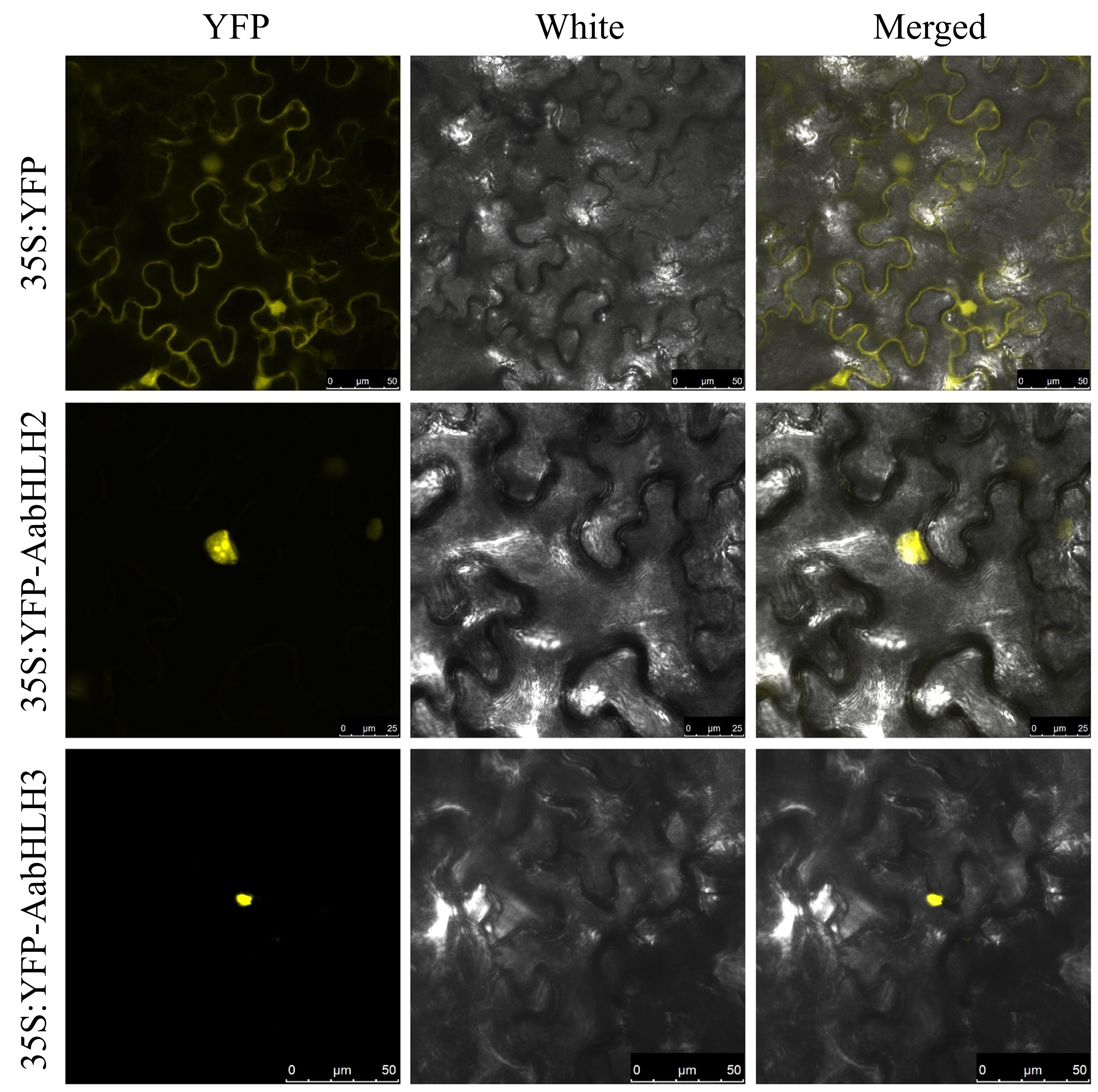

Supplement: Supplementary file 2 [file Image_1.JPEG]

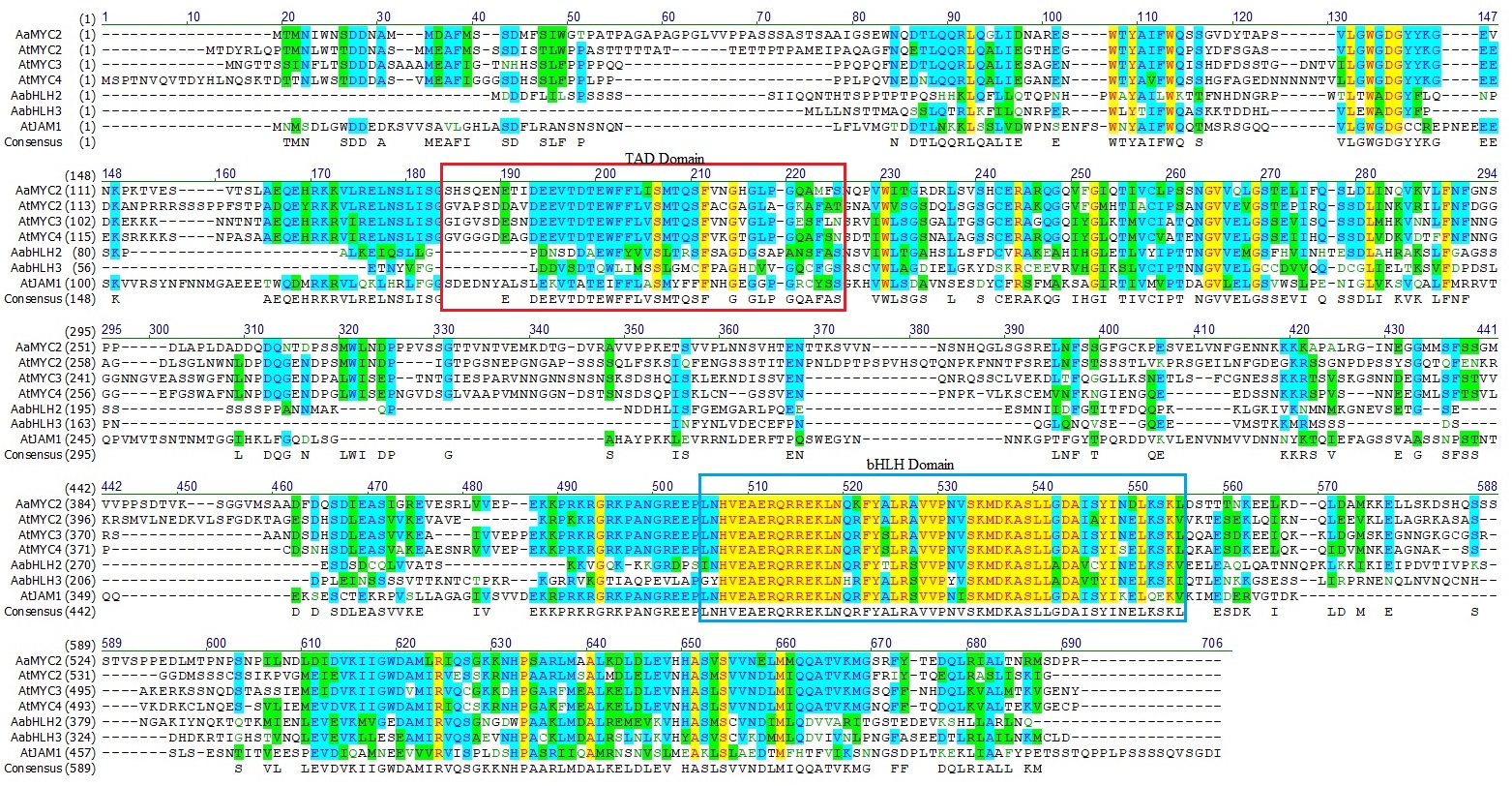

Supplement: Supplementary file 3 [file Image_2.JPEG]
